# Establishment and Comparison of Combining Disease and Syndrome Model of Asthma with “Kidney Yang Deficiency” and “Abnormal Savda”

**DOI:** 10.1155/2013/658364

**Published:** 2013-04-15

**Authors:** Bei Li, Qing-li Luo, Mammat Nurahmat, Hua-liang Jin, Yi-jie Du, Xiao Wu, Yu-bao Lv, Jing Sun, Muhammadjan Abduwaki, Wei-yi Gong, Jing-cheng Dong

**Affiliations:** ^1^Department of Integrative Medicine, Huashan Hospital, Fudan University, Shanghai 200040, China; ^2^Xinjiang Uighur Medical Training College, Wada, Xinjiang 848000, China

## Abstract

The study was the first time to establish and compare two rat models of two common syndromes: Kidney Yang Deficiency syndrome (KYDS) in traditional Chinese medicine (TCM) and abnormal savda syndrome (ASS) in traditional Uighur medicine (TUM). Then, we also established and evaluated rat models of combining disease and syndrome models of asthma with KYDS or ASS. Results showed that usage of the high dose of corticosterone (CORT) injection or external factors could successfully establish the KYDS or ASS rat models, and the two models had similar changes in biological characterization, abnormal behaviors, dysfunction of hypothalamic-pituitary-target organ axes (HPTOA), and sympathetic/parasympathetic (S/P) nerve system but varied in different degrees. The rat models of combining disease and syndrome of asthma with KYDS or ASS had either pathological characteristics of asthma such as airway hyperresponsiveness (AHR), airway inflammation, airway remodeling, which were more serious than allergy exposure alone, or the syndrome performance of Kidney Yang Deficiency in TCM and abnormal savda in TUM. These findings provide a biological rationale for further investigation of combining disease and syndrome model of asthma as an effective animal model for exploring asthma based on the theory of traditional medicine.

## 1. Introduction

Recently, asthma has become one of the most common health problems in the world, especially within industrialized societies [1–3], where underlying mechanisms are not yet completely understood. Increasing evidence suggests that asthma is a chronic inflammatory disorder of the airways in which many cells and cellular elements play a role [4] and is characterized by the imbalance of helper T cells (Th) 1/Th2 cytokines and dominant in Th2 cytokines [5]. However, the hypothesis that immune factors lead to airway inflammation cannot show the whole picture of asthma. Recently, the anti-inflammatory effect of endogenous glucocorticoids released by the activated hypothalamic-pituitary-adrenal axis (HPAA) attracts scientists' attention. A low HPAA activity in allergic patients has been reported in a large number of clinical trials [6, 7]. Initially, the main interest of researchers was concentrated on the HPAA of asthmatics that were on long-term treatment with inhaled corticosteroid (ICS); subsequently, a growing number of studies recognized that asthmatic patients not treated with ICS were also likely to have an attenuated activity and/or responsiveness of their HPAA [8, 9]. Moreover, researchers found that asthma was closely related to neuroendocrine-immune (NEI) network dysfunction [10, 11]. 

Traditional Chinese medicine (TCM) and traditional Uighur medicine (TUM) have their own cognitions, theories, and treatments for asthma. In the theory of TCM, Kidney Yang Deficiency syndrome (KYDS) is one of the most common syndromes seen in asthmatics and it has been found that HPAA dysfunction is the essential characteristic of KYDS [12, 13]. Moreover, studies found that KYDS may run through the entire process of asthma [14–17]. In TUM, there is also a common syndrome called abnormal savda syndrome (ASS), which is the main cause of complex diseases like asthma. Studies show that HPAA dysfunction may be the foundation and essence of ASS, which is the main syndrome in severe asthma [18, 19]. In a word, both of KYDS and ASS are common syndromes in asthmatics; moreover, researchers foud that they are both relevant to dysfunction of HPAA.

Due to the dysfunction of HPAA, we speculate that the anti-inflammatory effect of endogenous glucocorticoids in asthma patients with KYDS or ASS would be weaker than those patients without KYDS or ASS; thus, the symptoms of asthma would be more severe than those without KYDS or ASS. For a thousand years, traditional medicine has built a therapeutics system to relieve and cure asthma [20–22]. In order to certify and clarify the efficacy of therapies in traditional medicine, the work of establishing proper and optimal animal models plays an essential role in elucidating pathogenesis of different syndromes in different traditional medicine theories and exploring the traditional Chinese medicine and Uighur medicine to better prevent and treat asthma. Given these considerations, our principal aim was to establish KYDS and ASS rat models on basis of preliminary studies; then, the above rat models were combined with antigen-exposed, and KYDS-asthma (KYDSA), ASS-asthma (ASSA) rat models were therefore set up. We compared different models in aspects of general state, behaviors, hypothalamic-pituitary-target organ axes (HPTOA) and sympathetic/parasympathetic (S/P) nerve system function, airway hyperresponsiveness (AHR), airway inflammation, and airway remodeling. This may help us in clarifying the scientific basis of KYDS or ASS and designing novel animal model for exploring asthma based both on TCM and TUM.

## 2. Materials and Methods

### 2.1. Experimental Animals and Groups

 60 specific pathogen-free male Sprague Dawley (SD) rats (aged 6–8 wk, weighed 180–200 g, 5 per cage) were purchased from Shanghai SLAC Co. (Shanghai, China) and used in this study. All studies were performed in accordance with the recommendations of the Guide for the Care and Use of Laboratory Animals of Fudan University of Chinese Medicine. The protocol was approved by the Animal Experimental Ethical Committee of Fudan University of Chinese Medicine. All efforts were made to ameliorate suffering of animals. SD rats were allowed to acclimate to their new environment for 1 wk before experiment and were maintained on a 12 h light/dark schedule (lights on at 6:00 a.m.) with food and water available ad libitum. Then, the total of 60 male SD rats were randomly assigned to six groups (*n* = 10 per group): A, normal control group (NC group); B, sensitized and antigen challenged (asthma control group, AC group); C, high dose of corticosterone (CORT) injected (KYDS group); D, multiple factors experienced (ASS group); E, high dose of CORT injected, sensitized, and antigen challenged (KYDSA group); F, multiple factors experienced, sensitized, and antigen challenged (ASSA group).

### 2.2. Ovalbumin-(OVA-) Induced Asthmatic Model

 The AC group rats were sensitized and challenged by OVA (Sigma Chemical Co., St. Louis, MO, USA) according to the modified protocol reported previously [23]. Briefly, on day 1, rats received an intraperitoneal injection of 1 mL of 10% OVA (100 mg OVA in 1 mL normal saline) mixed with 100 mg of aluminum oxyhydrogen (Sigma Chemical Co., St. Louis, MO, USA) and 5 × 10^9^ heat-killed *Bordetella pertussis bacilli*, which were kindly donated by the National Vaccine and Serum institute. Two weeks after the sensitization, the rats inhaled 1% OVA for 30 min through an ultrasound aerification inhaler (Yisheng Technology Co. Ltd, Shanghai, China) in an exposure chamber (50 cm × 30 cm × 25 cm) for 14 successive days.

### 2.3. KYDS and ASS Rat models

 The KYDS group received 5 mg/kg exogenous CORT (Sigma Chemical Co., St. Louis, MO, USA) dissolved in olive oil (Argolis, Greece) by means of multipoint subcutaneous injection for 30 successive days [24, 25]. According to the theory of TUM, multiple factors including reared in a dry and cold environment, given dry and cold food, experienced chronic intermittent plantar electric shock, forced swimming, and fixed brake were used to establish the ASS group for 21 successive days. Briefly, rats were put in the intelligent artificial climate chamber (Ningbo Jiangnan instrument factory, China) imitating dry and cold environment, in which the temperature was set to 6 ± 1°C and the relative humidity was 25% ~ 32% during 11:00 a.m.–9:00 p.m. at the modeling days. The rats were fed special chow composed of 150 mg of coriander seed and 150 mg of barley in per kilogram standard rodent chow. In the theory of TUM, the nature of coriander seed and barley was dry and cold. The special chow was made in granules. The rats were provided 250 mg of special chow and 500 mL of water per cage from 2:00 p.m. to 10:00 a.m. of next day during the modeling process. Before 11 a.m., some rats were firstly given chronic intermittent plantar electric shock in the small animals jumping instrument (JXDT-II, Hinman Science and Education Equipment Co., Ltd., Shanghai, China): the first week rats experienced an electric shock under the voltage of 35 V for 30 min per day, the second week under the voltage of 40 V for 35 min per day, and the last week under the voltage of 45 V for 45 min per day in order to avoid rats adaption to the same stimulation. Meanwhile, some rats were firstly put in a self-made plexiglass cylindrical-shaped barrel with a volume of 100 cm × 20 cm × 20 cm to undergo forced swimming for 5 min, and some rats were fixed in rat fixers (Buxco, USA) to brake for 20 min. In a word, each rat every day, respectively, experienced electric shock, forced swimming, and brake during modeling process. The NC group was bred under regular laboratory conditions, with a controlled room temperature and a 12/12-hour light-dark cycle with free access to standard rodent chow and water. 

### 2.4. KYDSA and ASSA Rat Models

**Figure 1:**
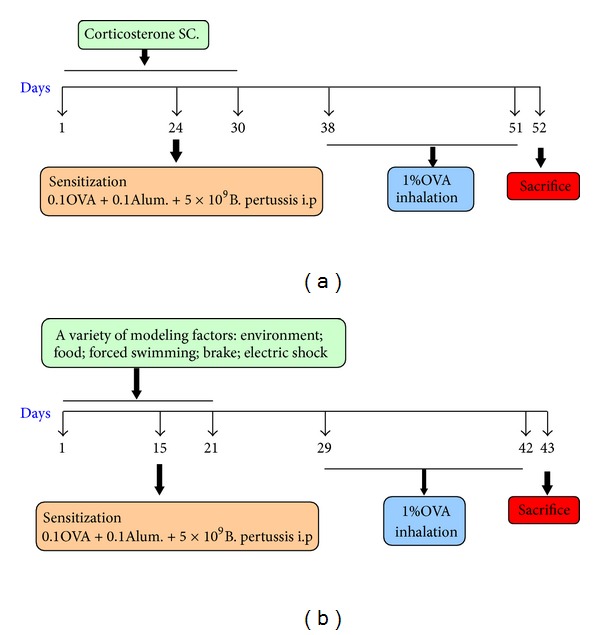
Rat models of combined KYDS or ASS with OVA sensitization and challenge.

The schedule of the KYDSA group is presented in Figure 1(a) and briefly summarized: days 1–30, high dose of CORT (multi-point subcutaneous injection); day 24, sensitization (ip); days 38–51, antigen challenge (inhalation); day 52, sacrifice. The schedule of the ASSA group is presented in Figure 1(b) and briefly summarized: days 1–21, multiple factors (environmental and dietary change, forced swimming, brake, electric shock); day 15, sensitization (ip); days 29–42, antigen challenge (inhalation); day 43, sacrifice. And the method of allergy sensitization and challenge was consistent with the AC group.

### 2.5. Observation of General State

During the process of modeling, general state including mood, hair, behaviors, body weight, food and water intake, and urine and stool condition was recorded every day. Food and water intake were calculated by the following formulas:

(1) food intake per 100 g body weight=250 (g)−remaining food weight (g)body weight (g)×100;water intake per 100 g body weight=500 (mL)−remaining water volume  (mL)body weight (g)×100.

### 2.6. Open-Field Test (OFT) and Sucrose Preference Test (SPT)

The OFT allows for the evaluation of general locomotor and exploratory behavior of rats [26]. The open-field apparatus was square shaped (100 cm × 100 cm × 50 cm) with walls made of black plastic and the floor painted black and divided into 25 equal sectors by white lines. Approximately on the morning immediately following the final day of modeling, all rats were acclimatized to the test room for 1 h. Each rat was placed in the center of the open field and behaved freely for 5 min; its behavior was recorded using a video camera placed above the open field. As indexes of ambulatory counts (number of individual horizontal movements registered when the mice walked on all four feet) and vertical counts (rearing counts registered when rats' body inclined vertically with hind paws on the floor and forepaws on the wall of the activity field) as well as the total across counts were evaluated and recorded. Moreover, the number of fecal boli was counted at the end of each trial. In order to reduce anxiety caused by the environment, low-level illumination was used throughout the experiment. After the test of one rat, the chamber was wiped by 30% alcohol in order to avoid the interference between different rats. The test was performed in the same room where the experimental animals were housed [27]. The intake of water and sucrose solution (1%) was measured as an operational index of anhedonia (reduced responsiveness to a pleasurable stimulus). The SPT was performed as described previously [28], with minor modifications. Before the test, the rats were trained to adapt to sucrose solution (1%, w/v) by placing two bottles of sucrose solution in each cage for 24 h; then one of the bottles was replaced with water for 24 h. After the adaptation procedure, the rats were deprived of water and food for 24 h. The SPT was conducted at 9:00 a.m. The rats were housed in individual cages and given free access to the two bottles containing 100 mL of sucrose solution (1%, w/v) and 100 mL of water, respectively. After 1 h, the volumes of consumed sucrose solution and water were recorded and the sucrose preference was calculated by the following formula:

(2) sucrose preference =sucrose consumptionwater consumption + sucrose consumption×100%.

### 2.7. Measurement of Thymus, Spleen, Adrenal, Thyroid, and Testicular Organ Index

Thymus, spleen, adrenal glands, thyroid, and testicular glands were removed, carefully trimmed, weighed, and respective organ index and calculated to evaluate possible atrophic and/or hyperplastic effects due to different modeling factors:

(3) organ index = organ weight  (g)body weight  (g)×100%.

### 2.8. Measurement of AHR by Buxco's Modular and Invasive System

AHR was assessed directly by measuring changes in pulmonary resistance response to increasing concentrations of inhaled methacholine (Mch, Sigma Chemical Co., St. Louis, MO, USA). Before Mch challenge and measurement of AHR, the rats were anesthetized with 2% pentobarbital sodium (60 mg/kg ip). Then a tracheostomy was made; the trachea was cannulated; the pleural spaces were opened; the rats were placed in a whole-body plethysmography chamber (Buxco, USA) for anesthetized animals; and the trachea was connected with the small animal ventilator [29]. The ventilator frequency was set to 120 r/min and the flow was adjusted to the maximum tidal volume of 0.6–1.5 mL. After a stable baseline airway pressure (<5% variation over 2.5 min) was reached, PBS and increasing concentrations of Mch (3.125, 6.25, 12.5, 25 mg/mL) in succession were administered via a jet nebulizer into the head chamber. Minimum values for airway resistance (RI) were determined and AHR was expressed as percentage change from the baseline value [30].

### 2.9. Analysis of Plasma Hormones to Evaluate HPTOA and S/P Nerve System Function

In order to diminish the circadian rhythm differences of hormone levels, plasma was collected at the end day of modeling by the means of eyeball and separated in a refrigerated centrifuge at 4°C, 2500 rpm for 10 min. Plasma was stored at −20°C until assays. After the process of collection plasma, animals were also alive. Levels of CORT, adrenocorticotropic hormone (ACTH), 3,5,3′-triiodothyronine (T_3_), thyroxine (T_4_), thyroid-stimulating hormone (TSH), estradiol (E2), testosterone (T), luteinizing hormone (LH), follicle stimulating hormone (FSH), cyclic adenosine monophosphate (c AMP), and cyclic guanosine monophosphate (c GMP) were measured by enzyme-linked immunosorbent assay (ELISA) kits (R&D Systems, Minneapolis, USA) following the manufacturer's instructions. Take CORT as an example, 10 *μ*L of plasma and 0.5 *μ*L of steroid displacement reagent was diluted in 990 *μ*L of assay buffer, completing a 100-fold dilution. Plates were coated with 100 *μ*L of anti-CORT overnight at 4°C then blocked with assay buffer for 2 h at room temperature. After two washes with PBS-Tween, 100 *μ*L of standards and supernatant samples were added and incubated overnight at 4°C. Plates were washed four times before 100 *μ*L/well of biotinylated anti-CORT was added. After 45 min at room temperature, plates were washed six times with PBS-Tween. After addition of 100 *μ*L/well of a 1 : 1000 dilution of avidin-peroxidase, plates were incubated at room temperature for 30 min. After eight washes, 100 *μ*L of substrate solution was added per well, and the enzymatic reaction was allowed to develop at room temperature. OD was measured at 405 nm on an ELISA plate reader. CORT concentrations were quantified by comparison to the standard curves. Samples were analyzed in duplicate in a single assay; threshold detection = 150 pg/mL; coefficient of variation limit = 9.6%; concentration expressed in pg/mL.

### 2.10. Bronchoalveolar Lavage Fluid (BALF) Preparation and Cytokines Analysis. 

Owning to the invasive pulmonary function test, the rats' tracheas were cannulated, and their chests were opened. BALF was performed three times (0.5 mL PBS/lavage) through the tracheal cannula [31]. The recycling lavage aliquots about 1ml were pooled and centrifuged. The supernatant was stored at −20°C for cytokine detection. The levels of interleukin (IL)-2, IL-5, IL-6, IL-10, IL-13, granulocyte-macrophage colony-stimulating factor (GM-CSF), and interferon-*γ* (IFN-*γ*) in BALF were detected using Bio-Plex Suspension Array System (R&D Systems, Minneapolis, USA).

### 2.11. Measurement of Cytokines in Serum

After BALF was collected, serum was collected by the means of abdominal aorta and separated in a refrigerated centrifuge at 4°C, 2500 rpm for 10 min. Serum was stored at −20°C until assays. Levels of IL-2, IL-5, IL-6, IL-10, IL-13, GM-CSF, and IFN-*γ* in serum were measured using Bio-Plex Suspension Array System according to the manufacturer's instructions.

### 2.12. Lung Histological Analysis

The left lungs were removed by dissection and fixed in 4% paraformaldehyde. Lung tissues were sectioned, embedded in paraffin, and cut into 3 *μ*m sections. Tissue sections were then stained with hematoxylin-eosin (H&E) for general morphology [32] and with periodic acid-Schiff (PAS) for the identification of goblet cells in the epithelium [33]. Quantitative analyses of cell infiltration and mucus production were performed blindly as previously described [34]. Inflammatory changes were graded using a semiquantitative scale of 0–5 (Table 1) for perivascular eosinophilia, peribronchiolar eosinophilia, epithelial damage, and oedema [35]. MASSON staining was used here to detect collagen deposition in the lung tissues according to previous reports [36, 37]. After staining, the collagen area on the basal membrane of the airway in the lung tissues was analyzed by optical microscope and Nikon digital photography integrated system (Nikon, Japan) using the affix color model. The result was expressed as Wac/Pbm (*μ*m^2^/*μ*m) (collagen area on the basal membrane of airway/the length of the airway epithelial).

### 2.13. Statistical Analysis

Data were expressed as mean ± standard deviation (SD). SPSS 17.0 software was used for statistical analysis. Differences between mean values of normally distributed data were assessed by one-way analysis of variance (ANOVA) followed by Bonferroni's posthoc comparison tests. For comparison of two groups, a student's *t*-test was used. Statistical differences were considered significant at *P* < 0.05.

## 3. Results

### 3.1. Body Weight, Food and Water Intake in Different Groups

**Figure 2:**
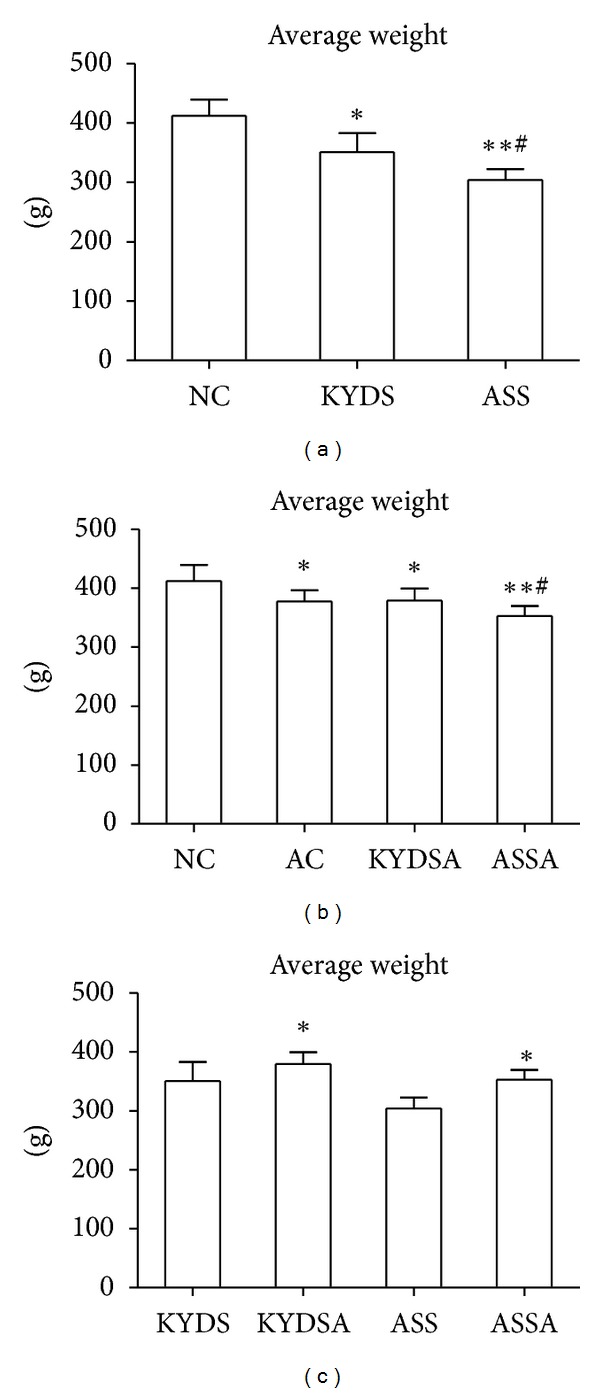
Body weight was measured and recorded before and after experiment. There was no significant difference among six groups before the experiment. However, when rats were sacrificed, the body weight gain was statistically different. Data are expressed as mean ± SD, *n* = 10 rats per group. (a): **P < 0.01, *P < 0.05, versus the NC group; ^#^ *P* < 0.05, versus the KYDS group. (b): **P < 0.01, *P < 0.05, versus the NC group; ^#^ *P* < 0.05, versus the AC group. (c): *P < 0.05, versus the KYDS group or the ASS group.

**Figure 3:**
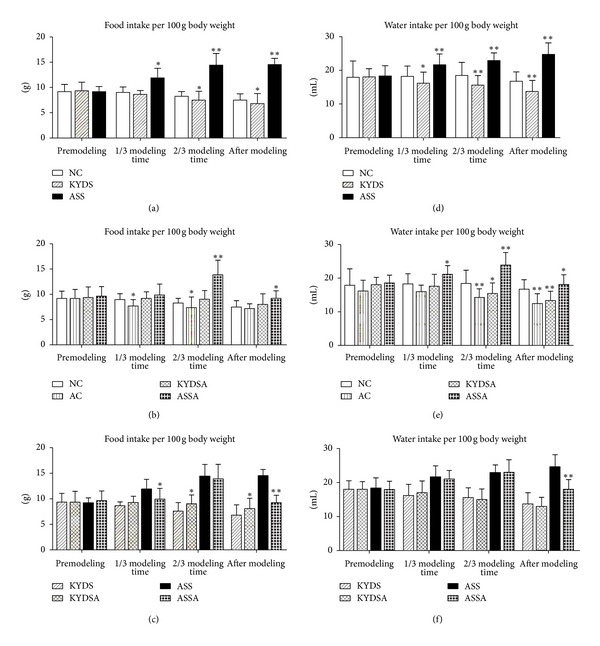
Food and water intake were calculated during the three point time of the whole experiment process. Before the experiment, there was no significant difference in food and water intake among six groups. Data are expressed as mean ± SD, *n* = 10 rats per group. (a) and (d): **P < 0.01, *P < 0.05, versus the NC group. (b) and (e): **P < 0.01, *P < 0.05, versus the NC group. (c) and (f): *P < 0.05, versus the KYDS group or the ASS group.

We firstly tested if there was a difference in general state among different models. Firstly, rats in the KYDS and ASS with or without OVA-exposed groups had irritable mood, dry and dim hair, fatigue and sleepiness, quantity of smelly urine, and dark and red tongue. OVA-exposed Rats had sneezing, shortness of breath, curled, arched, cyanosis of lips, sputum, and frequent washing. Secondly, there was no significant difference in body weight among groups before the experiment. However, as illustrated in Figure 2, when rats were at sacrifice, body weight in the AC, KYDS, ASS, KYDSA and ASSA groups was significantly decreased compared to the NC group (*P* < 0.05, *P* < 0.01). The body weight gain in the ASS group was lower than that in the KYDS group (*P* < 0.05, Figure 2(a)). Moreover, body weight had been slightly increased in the KYDSA, and ASSA groups during allergy exposure compared to those groups without allergy exposure (*P* < 0.05, Figure 2(c)), but there was still significant difference with the NC group (*P* < 0.05, Figure 2(b)). As showed in Figure 2(b), body weight in the ASSA group was significantly decreased compared to the KYDSA group (*P* < 0.05). As seen in Figure 3, food and water intake was markedly increased in the ASS with or without allergy exposure groups but decreased in the KYDS with or without allergy exposure groups compared to the NC group (*P* < 0.05, *P* < 0.01). Similarly to the KYDS group, food and water consumption significantly declined in the AC group in contrast to the NC group (*P* < 0.01).

### 3.2. The OFT and SPT in Different Groups

**Figure 4:**
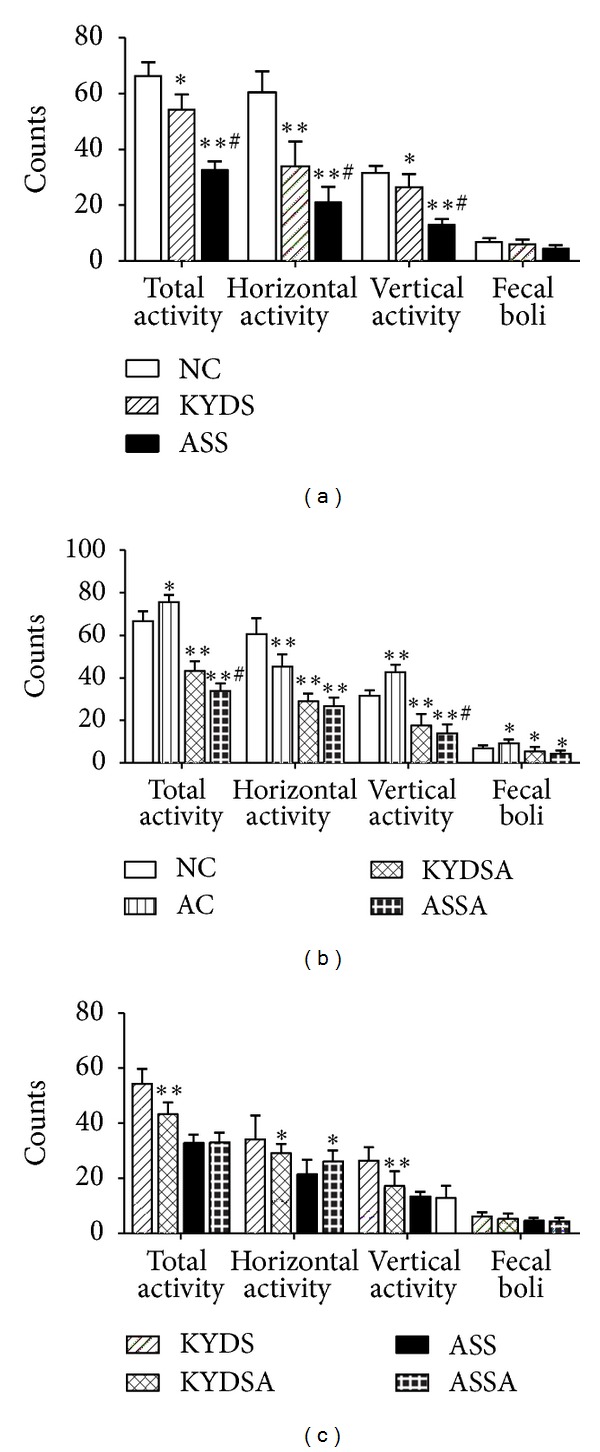
In the open field test, spontaneous activity was evaluated when rats were response to a new environment. Data are expressed as mean ± SD, *n* = 10 rats per group. (a): **P < 0.01, *P < 0.05, versus the NC group; ^#^ *P* < 0.05, versus the KYDS group. (b): **P < 0.01, *P < 0.05, versus the NC group; ^#^ *P* < 0.05, versus the KYDSA group. (c): **P < 0.01, *P < 0.05, versus the KYDS group or the ASS group.

**Figure 5:**
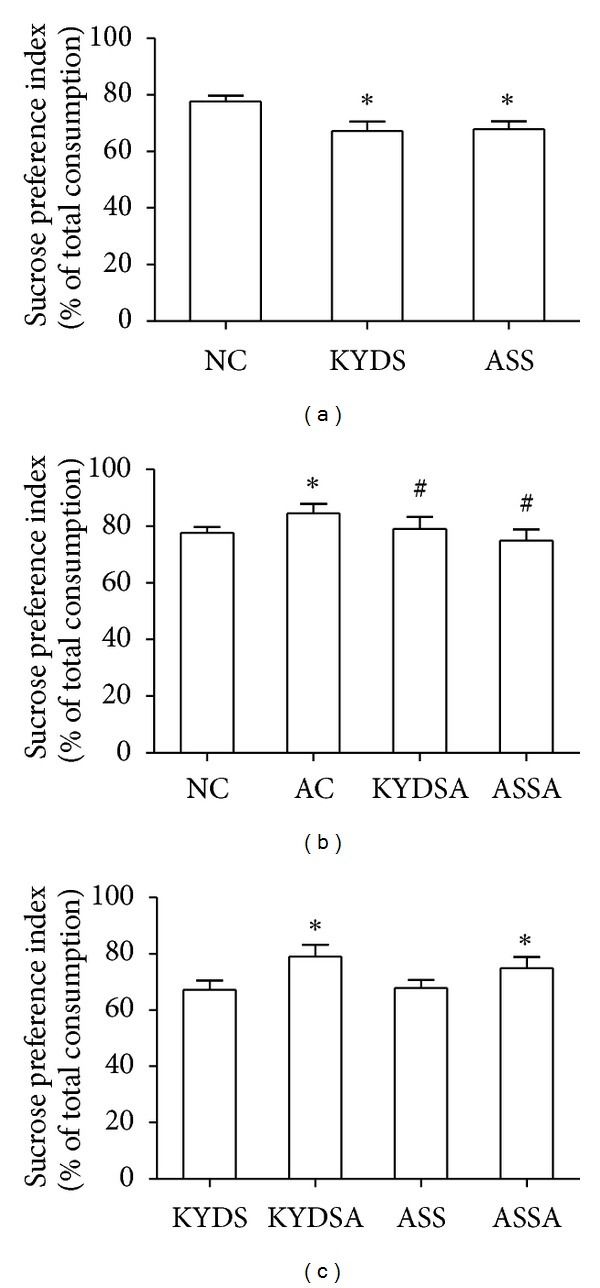
In the sucrose preference test, total water intake, sucrose water intake and pure water intake were recorded, and then the index of sucrose preference was calculated. Data are expressed as mean ± SD, *n* = 10 rats per group. (a): *P < 0.05, versus the NC group. (b): *P < 0.05, versus the NC group; ^#^ *P* < 0.05, versus the AC group. (c): *P < 0.05, versus the KYDS group or the ASS group.

We performed two behavioral tests to determine if rats subjected to different modeling factors demonstrated common behavioral phenotypes of affective dysfunction. As showed in Figure 4(a), the total cross counts, horizontal and vertical counts, were significantly diminished in the KYDS group (*P* < 0.05) and especially in the ASS group (*P* < 0.01) compared to the NC group. However, rats in the AC group sharply increased the activities in the OFT (Figure 4(b)). Moreover, rats with OVA exposure had markedly decreased spontaneous activities compared to those without OVA exposure in the OFT (Figure 4(c)).  Figure 5 reported changes of sucrose solution intake after modeling among groups.  Figure 5(a) showed that average sucrose intake sharply decreased in the KYDS and ASS groups (*P* < 0.05).  Figure 5(b) demonstrated that the AC group had significantly increased sucrose intake (*P* < 0.05) compared to the NC group but decreased in the KYDSA and ASSA groups in contrast to the AC group (*P* < 0.05). Conversely, sucrose intake was lower in the KYDS and ASS groups than those with OVA exposure (*P* < 0.05). The SPT indicated that rats in the KYDS and ASS with or without allergy exposure had clear signs of a depression-like state, but anxiety-like state in the AC rats.

### 3.3. Organ Index of Thymus, Spleen, Adrenal, Thyroid, and Testicular Glands in Different Groups

**Figure 6:**
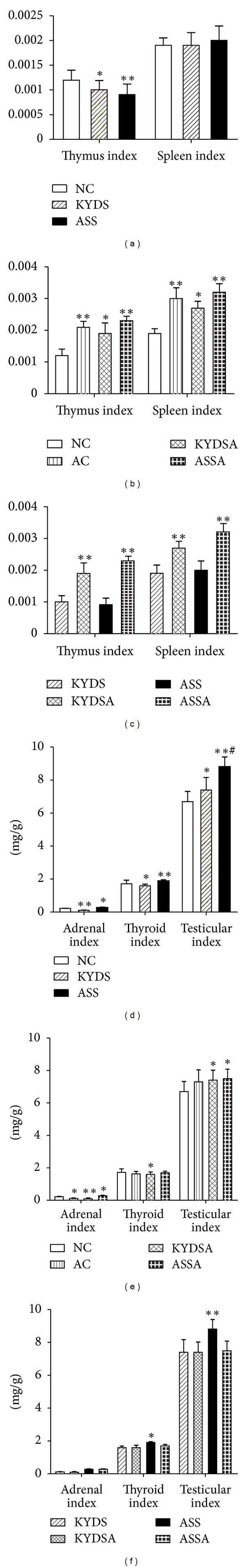
In order to evaluate possible atrophic and/or hyperplasia effects on immune organs and HPTOA glands due to modeling, thymus, spleen, adrenal, thyroid and testicular gland were removed, carefully trimmed, and weighed after rats were sacrificed. Data are expressed as mean ± SD, *n* = 10 rats per group. (a) and (b): **P < 0.01, *P < 0.05, versus the NC group. (c): **P < 0.01, versus the KYDS group or the ASS group. (d): **P < 0.01, *P < 0.05, versus the NC group; ^#^ *P* < 0.05, versus the KYDS group. (e): **P < 0.01, *P < 0.05, versus the AC group. (f): *P < 0.05, versus the KYDS group or the KYDSA group or the ASSA group.

Figure 6(a) illustrated that rats in the KYDS and ASS groups with induced thymus atrophy indicated immune suppression (*P* < 0.05, *P* < 0.01); however, showed in the Figures 6(b) and 6(c), rats with OVA exposure had induced thymus and spleen hyperplasia compared to those without OVA exposure which suggested immune activation due to allergy exposure (*P* < 0.01). Figures 6(d) and 6(e) demonstrated that the KYDS and KYDSA groups manifested adrenal and thyroid atrophy (*P* < 0.05), but testicular hyperplasia (*P* < 0.05), and above three glands had hyperplasia in the ASS and ASSA groups (*P* < 0.05) in contrast to the NC group. Moreover, the adrenal gland from the AC group was atrophy (*P* < 0.05) compared to the NC group, but there was no significant difference in thyroid and testicular glands. As seen in Figure 6(f), the ASS group was more obvious in thyroid and testicular gland hyperplasia than other groups (*P* < 0.05).

### 3.4. Analysis of HPTOA and S/P Nerve System Function

**Figure 7:**
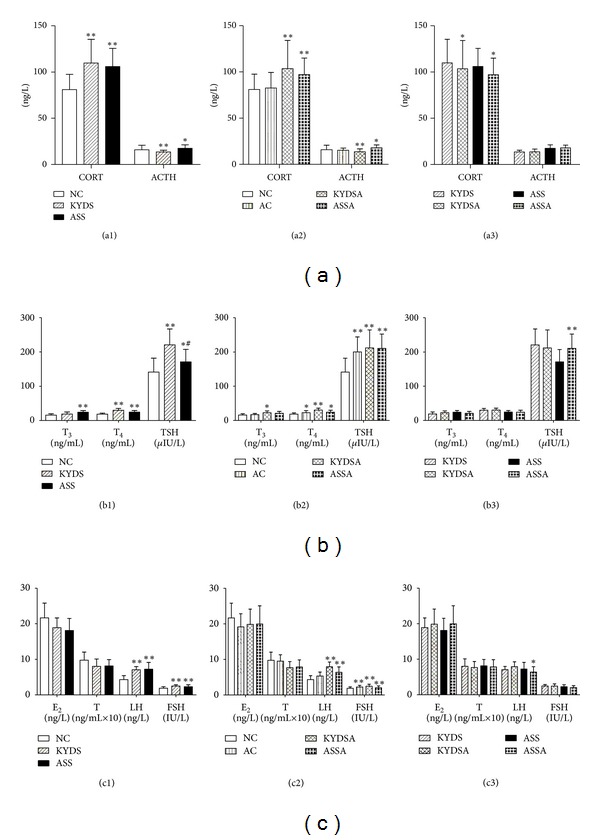
Function of HPTOA was evaluated by the analysis of plasma hormones. Data are expressed as mean ± SD, *n* = 10 rats per group. (a1): **P < 0.01, *P < 0.05, versus the NC group. (a2): **P < 0.01, *P < 0.05, versus the NC group or the AC group. (a3): *P < 0.05, versus the KYDS group or the ASS group. (b1): **P < 0.01, *P < 0.05, versus the NC group; ^#^ *P* < 0.05, versus the KYDS group. (b2): **P < 0.01, *P < 0.05, versus the NC group. (b3): *P < 0.05, versus the ASS group. (c1): **P < 0.01, versus the NC group. (c2): **P < 0.01, *P < 0.05, versus the NC group. (c3): *P < 0.05, versus the ASS group.

**Figure 8:**
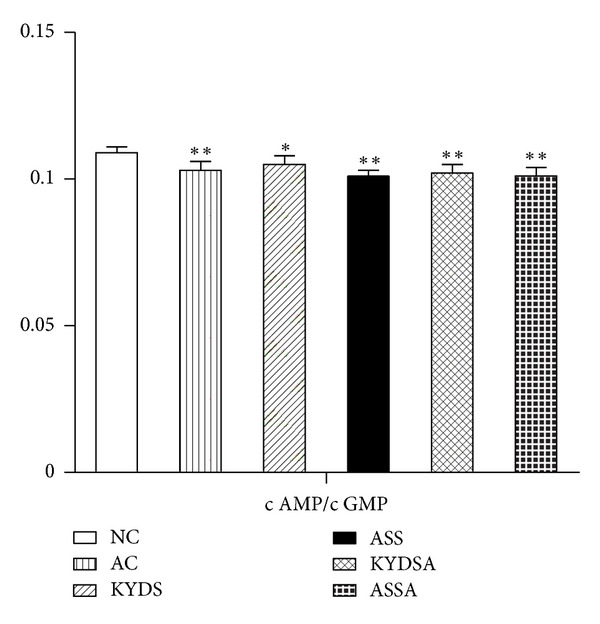
Dysfunction of S/P nerve system was induced in the five modeling groups compared to the NC group. Data are expressed as mean ± SD, *n* = 10 rats per group. **P < 0.01, *P < 0.05, versus the NC group.

To determine the function of HPOTA and S/P nerve system due to modeling, we analyzed plasma hormones and second messengers like c AMP and c GMP.  Figure 7 showed the levels of CORT, T_4_, TSH, LH, and FSH that notably increased in plasma in the KYDS and KYDSA groups (*P* < 0.01), but the level of ACTH significantly decreased (*P* < 0.05) compared to the NC group, which demonstrated that those groups had the dysfunction of HPTOA. Moreover, the ASS and ASSA groups activated the three HPTOA. Although the level of CORT went down and ACTH dropped up in the AC group, there was no significant difference with the NC group.  Figure 8 illustrated that the ratio between c AMP and c GMP was remarkably reduced in the other five groups compared with the NC group (*P* < 0.05, *P* < 0.01), which indicated that those groups had a dysfunction of the S/P nerve system.

### 3.5. AHR Assessment

**Figure 9:**
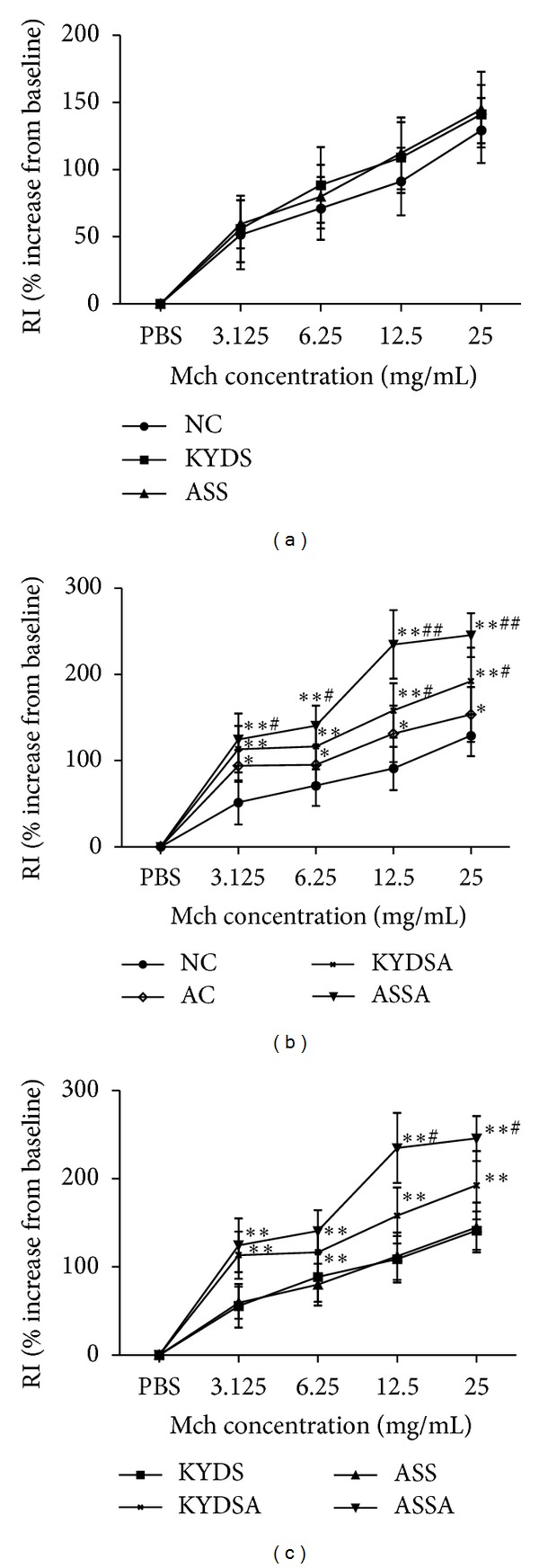
Airway hyperresponsiveness (AHR) was assessed more directly by measuring changes in airway resistance in response to increasing concentrations of inhaled methacholine (Mch). Data are expressed as mean ± SD, *n* = 10 rats per group. (b): **P < 0.01, *P < 0.05, versus the NC group; ^#^ *P* < 0.05, versus the AC group. (c): **P < 0.01, *P < 0.05, versus the KYDS group or the KYDSA group or the ASSA group; ^#^ *P* < 0.05, versus the KYDSA group.

As showed in Figure 9, OVA-exposed rats developed AHR compared to those without allergy exposure, typically reflected by high RI in response to increasing concentrations of Mch (*P* < 0.05, *P* < 0.01). The KYDSA group had higher RI than the AC group (*P* < 0.05) but was lower than the ASSA group (*P* < 0.05) at the Mch concentrations of 12.5 mg/mL and 25 mg/mL. Moreover, the change of RI was significantly increased in the ASSA group compared to the AC group at every concentration of Mch (*P* < 0.05). 

### 3.6. Evaluation of the Levels of Th1 and Th2 Cytokines in BALF and Serum

**Figure 10:**
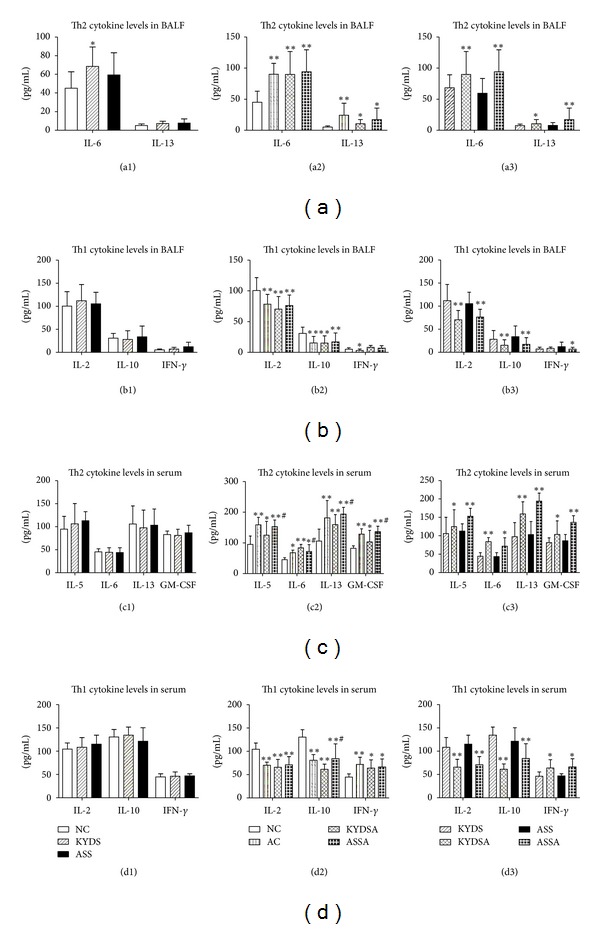
Analysis of pro-inflammatory and anti-inflammatory cytokines in bronchoalveolar lavage fluid (BALF). BALF was harvested during the 24 h after last OVA challenge. Data are expressed as mean ± SD, *n* = 10 rats per group. (a1): *P < 0.05, versus the NC group; (a2) and (b2): **P < 0.01, *P < 0.05, versus the NC group; (a3) and (b3): **P < 0.01, *P < 0.05, versus the KYDS group or the ASS group. (c2) and (d2): **P < 0.01, *P < 0.05, versus the NC group; ^#^ *P* < 0.05, versus the KYDSA group. (c3) and (d3): **P < 0.01, *P < 0.05, versus the KYDS group or the ASS group.

Figures 10(a) and 10(b) reported that the levels of Th2 inflammatory cytokines like IL-6 and IL-13 were sharply increased in the OVA-exposed groups (*P* < 0.05, *P* < 0.01), but Th1 cytokines like IL-2 and IL-10 were significantly decreased compared to non-OVA-exposed groups (*P* < 0.01). Due to the sensitivity of detection equipment, the levels of IL-5 and GM-CSF had not been measured and included in the statistics, but there was an upward trend in the groups of AC, KYDSA, and ASSA (data not shown here). Figures 10(c) and 10(d) demonstrated that OVA exposure were sharply increased the levels of Th2 inflammatory cytokines such as IL-5, IL-6, IL-13 and GM-CSF in serum (*P* < 0.01), but Th1 cytokines like IL-2 and IL-10, were significantly decreased (*P* < 0.01) compared to those without OVA exposure, which illustrated that rats experienced OVA challenge had induced imbalance of Th1/Th2 cytokines in serum. Moreover, the levels of IL-5, IL-13, and GM-CSF were higher in the ASSA group than those in KYDSA group (*P* < 0.05), and the level of IL-10 was lower in the KYDSA group than it in the AC group (*P* < 0.05). The tendency of cytokine levels' changes in serum was consistent with those in BALF. Moreover, though linear correlation analysis, it suggested that the level of IL-6 in serum showed a significant positive correlation with the level in BALF (*r* = 0.353, *P* = 0.013).

### 3.7. Lung Histopathological Analysis

**Figure 11:**
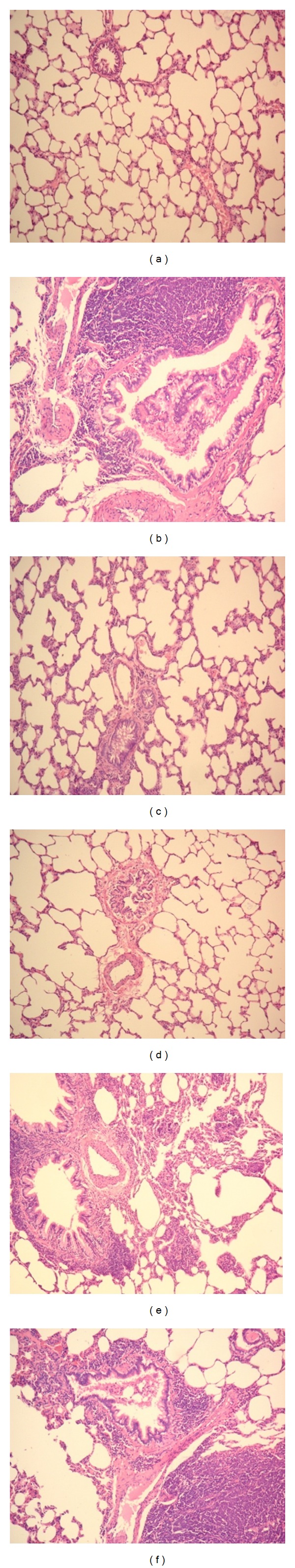
Detection of pulmonary inflammation according to the lung pathology. Lung tissues were obtained on the day after the last OVA challenge. Tissues were stained with hematoxylin and eosin (H&E, 200x). (a): NC group; (b): AC group; (c): KYDS group; (d): ASS group; (e): KYDSA group; (f): ASSA group. Note: Lung tissues were stained by hematoxylin and eosin (H&E), periodic acid-schiff (PAS) and masson, and scores mirrored to airway inflammation and airway remodeling were performed blindly by two researchers. Data are expressed as mean ± SD, *n* = 10 rats per group. **P < 0.01, *P < 0.05, versus the NC group; ^#^ *P* < 0.05, vs the AC group.

As showed in Table 1 and Figure 11, inflammatory cells especially eosinophils and lymphocytes infiltration in the peribronchial and perivascular areas (H&E staining), mucus overproduction and goblet cell hyperplasia (PAS staining), and emerge of collagen deposition (MASSON staining) were observed in these OVA-exposed rats' lungs (*P* < 0.01). Moreover, the degree of inflammatory cells infiltration in the peribronchial and perivascular areas (*P* < 0.05) and emerge of collagen deposition (*P* < 0.05) in the KYDSA group were more serious compared with the AC group. The scores of inflammatory cells infiltration (*P* < 0.05) and collagen deposition were higher in the ASSA group compared to the AC group.

## 4. Discussion

KYDS and ASS are, respectively, typical syndromes in TCM and TUM, and numerous studies have been carried out in order to explore the material basis of these two syndromes. Since 1950s, our team began to study the essence of KYDS and proved that the KYDS tended to involve the dysfunction of three target gland axes [38–40] and a key link of it may be in the level of hypothalamus [41, 42]. Since the beginning of the 1990s, we paid our attention to the theory of NEI [43], by rats injected using high doses of CORT, whose function of HPAA and immune system were suppressed by exogenous CORT, as the model of KYDS. After external CORT, neurotransmitters such as norepinephrine, dopamine, and serotonin, levels were increased. By utilizing a formula to supplement Kidney Yang, all those levels turned better [44]. The result supported the views that regulatory center of KYDS might lie in the level of hypothalamus and that its essence was closely related to NEI network. Upur et al. had utilized external multiple factors to imitate the pathogenic factors of ASS and established murine and rat model [45]. Based on this model, they found that the foundation of ASS was closely related to the dysfunction of immune system [46, 47], HPAA [18, 48], and deletion of Ace gene [49]. Hence, the pathogeneses of KYDS and ASS were both closely related to dysfunction of the NEI network. The present study carried on establishing and comparison of the KYDS and ASS rat models and found that there were similarities and differences between them. Both of them showed abnormal biological appearance, such as body weight loss, irritable mood, dry and dim hair, fatigue and sleepiness, quantity of smelly urine, dark and red tongue, reduced spontaneous activity, and declined preference to sucrose water, those change being especially apparent in the ASS group. However, they were different in the following aspects: dropped appetite, thin and fetid stool in the KYDS group, conversely, increased food and water intake, dry and slender stool, and tongue with petechia but little coating in the ASS group. Furthermore, both of them had dysfunction of the immune system, HPTOA, and S/P nerve system; however, the degree and performance were different. The KYDS group manifested adrenal and thyroid atrophy, but testicular hyperplasia, and the levels of CORT, T_4_, TSH, LH, and FSH notably increased in plasma, but the level of ACTH significantly decreased. But above three glands was hyperplasia and the levels of CORT, ACTH, T_4_, TSH, LH, and FSH all increased in the ASS group. The degree of these changes was more conspicuous in the KYDS group. These results suggested that changes of biological characterization were more obvious in the ASS rat model, but the degree of NEI network dysfunction was more severe in the KYDS rat model. It is well accepted that the HPAA is a major part of the neuroendocrine system and its main role is to subserve the body's response to a stressor, physical or emotional, that disrupts the homeostatic balance of the organism [50]. In fact, the present study compared two different models of neuroendocrine distress. One was obtained by glucocorticoid injections (called KYDS) and another by external factors (called ASS); both of them activated the HPAA, which resulted in an increase in circulating corticosteroids. This study showed us that the internal or external neuroendocrine distress could activate the HPAA, especially internal distress.

Asthma as a chronic inflammatory disorder of the airways, inherent anti-inflammatory capacity of the HPAA, plays an important role in the pathogenesis and development of asthma. A low HPA axis activity in allergic patients has been reported in a large number of clinical studies [6–9]. Traditional medicine has a long history of human use and has a unique system of theory and diagnosis and treatment tools, which is quite different from modern medicine. TCM is found on theoretical basis such as the Yin and Yang theory and five elements theory and physiological and pathological basis such as organs and meridians, focusing on the overall concept and syndrome differentiation, diagnosing based on the combination of observation, listening, interrogation, and pulse taking. The theoretical basis of TUM is the Hilit theory including Savda, Belghem, Sapra, and Kan, which are regarded as the basic substances involved in physiological activity [51, 52]. KYDS and ASS as typical syndromes in TCM and TUM are common syndromes in asthma patients. Although corticosteroids and *β*_2_-agonists are effective in managing asthma symptoms, there is no curative therapy. There are also concerns regarding the side effects from chronic use of current drugs, particularly by children and the glucocorticoids-resistant asthmatic patients. In China, there are a number of antiasthma herbal formulas recorded in TCM or TUM textbooks and used in practice, but evidence-based research into their efficacy and mechanisms of efficacy is still in its infancy. In order to investigate the efficacy, safety, and consistency of the complementary and alternative medicine, providing proper and optimal animal models is very essential. In present study, we established animal models of combining asthma with syndromes. The results showed that airway inflammation, AHR, and airway remodeling were more serious in the KYDSA and ASSA groups than the allergy-exposed alone group. Interestingly, Ke et al.'s [53] clinical study also found that the inflammation and obstruction on the bronchi were more serious in asthmatic patients with ASS than those without ASS. The reason why we compared the KYDSA and ASSA rat models was that there were several similarities between KYDS and ASS, and we would like to see the similarities and differences between KYDSA and ASSA. The results showed that both of them indicated signs of deficiency performance, such as body weight loss, fatigue, curling and decreased activity, slowed reaction in the OFT, declined adaption to a new environment, and also dropped preference to sucrose water. However, the above performance was more serious in the ASSA model. Moreover, appetite increased in the ASSA model but dropped in the KYDSA model. Secondly, both of them had dysfunction of immune system, HPTOA, and S/P nerve system. The ASSA group had increased hormones and activated the three target glands axis function, but HPAA was significantly suppressed in the KYDSA model. The content of c AMP and the ratio between c AMP and c GMP was remarkably reduced in the two groups, but there was no significant difference between them. Finally, both of them not only had the performance of asthma attack such like sneezing, shortness of breath, curled, arched, cyanosis of lips, sputum, and frequent washing when rats were received OVA challenge but also had the characteristic of AHR, airway inflammation, and airway remodeling. However, the degree was slightly different. The KYDSA group was accompanied by the performance of KYDS in addition to asthma symptoms, but the ASSA group was accompanied by the symptoms of ASS. Airway resistance in the ASSA group was higher than the KYDSA group at the Mch concentration of 12.5 mg/mL and 25 mg/mL. The levels of Th2 cytokines like IL-13, GM-CSF in serum and IL-13 in BALF in the ASSA were higher than those in the KYDSA group, but the levels of Th1 cytokines like IL-2 and IL-10 were lower in the KYDSA group than those in the ASSA group. The results of lung pathology showed that the degree of inflammatory cell infiltration in the peribronchial and perivascular areas, mucus secretion and emergence of collagen deposition were more severe in the KYDSA group than those in the ASSA group, but the scores of epithelial damage and edema were higher in the ASSA group than those in the KYDSA group, where no significant difference might be relevant to small sample size. In a word, the KYDSA and ASSA were similar to some extent, with respect to severity of asthma and NEI network disorder, but different in degrees. The animal models of combined asthma with KYDS and ASS lead us to know the negative impact of stress, internal or external factors, on asthma. It is well recognized that stress contributes to exacerbations of asthma [54, 55]; the underlying mechanism is so complicated that it is not discussed in this paper.

## 5. Conclusion

In summary, the present study demonstrated that using the methods of high dose of CORT injection and external factors could establish the rat models of KYDS and ASS, respectively. Then, combined with OVA sensitization and challenge, we also set up rat models of combining asthma with syndromes. By comparing with different indicators, we found that the KYDS and ASS were similar to some extent, with respect to dysfunction of the immune system, HPTOA, and S/P nerve system, which indicated that there might be a similar material basis about these two syndromes. Moreover, asthma with KYDS and ASS was more serious than asthma without these syndromes. This study builds up five rat models to simulate patients in clinics: asthma, KYDS, ASS, KYDSA, and ASSA, and these rat models may promote exploring the efficacy and the mechanism of efficacy for traditional medicines like TCM and TUM. By comparing analogous syndromes in different traditional medicines, it may encourage establish a holistic medical system in the future. 

## Figures and Tables

**Table 1:** HE, PAS and MASSON staining were used to assess the severity of inflammation, epithelial damage, oedema, mucus secretion and collagen deposition in lung tissues.

Group	Inflammatory cells infiltration in the peribronchial and perivascular areas	Oedema	Epithelial damage	Mucus area	Collagen deposition
NC	1.25 (1–3)	0.25 (0-1)	0	4.81 ± 1.91	4.68 ± 0.78
AC	3.12 (2–5)**	2.75 (1–5)**	3.12 (2–5)**	14.66 ± 1.51**	12.69 ± 2.17**
KYDS	2.00 (1–3)	1.75 (1–3)*	1.75 (1–3)*	7.60 ± 0.36	8.67 ± 1.03
ASS	2.05 (1–3)	1.95 (1–5)*	1.06 (1-2)	8.16 ± 0.56	8.32 ± 0.78
KYDSA	4.50 (4-5)^∗∗#^	2.25 (1–5)**	2.25 (1–5)**	14.75 ± 1.83**	17.68 ± 1.53^∗∗#^
ASSA	4.01 (4-5)^∗∗#^	2.58 (1–5)**	3.02 (2–5)**	11.83 ± 1.62**	14.72 ± 1.47**

Lung tissues were stained by HE, PAS and MASSON, and scores mirrored to airway inflammation and airway remodeling were performed blindly by two researchers. The data is shown as average scores ± SD, *n* = 10. **P < 0.01, *P < 0.05, versus the NC group; ^#^ *P* < 0.05, versus the AC group.
